# Tuberculous Pleurisy Detected by Lung Ultrasound

**DOI:** 10.31662/jmaj.2025-0356

**Published:** 2025-12-26

**Authors:** Yuki Ohnishi, Yasuhiro Suyama, Kenichi Nakamura

**Affiliations:** 1 Department of Family Medicine, SUNY Upstate Medical University, Syracuse, New York, USA; 2 Department of Rheumatology, NTT Medical Center Tokyo, Tokyo, Japan; 3 Department of General Internal Medicine, Aso Iizuka Hospital, Fukuoka, Japan

**Keywords:** pleura, tuberculosis, tuberculous pleurisy, ultrasound

A previously healthy 37-year-old Japanese woman presented with a 6-day history of right flank muscle cramps followed by 4 days of fever. She also reported dyspnea on exertion and occasional mild dry cough, both with unknown onsets. Physical examination was unremarkable, including absence of chest pain exacerbation with deep inspiration and no pleural friction rub appreciated. Lung ultrasound was performed (Figure 1A and 1B). Thoracentesis revealed an exudative pleural effusion with lymphocytic predominance and an adenosine deaminase level of 81 U/L. An enhanced chest computed tomography was obtained (Figure 2). T-SPOT was negative. She was admitted upon presentation. Sputum culture obtained on the day of admission confirmed Mycobacterium tuberculosis complex after 15 days of incubation. We diagnosed tuberculous pleurisy. The patient was referred to a tuberculosis treatment center for further management, where combination therapy with isoniazid, rifampin, ethambutol, and pyrazinamide led to resolution of symptoms. Later, she disclosed that her father, who died when she was 10 years old, had been treated for tuberculosis.

**Figure 1.**
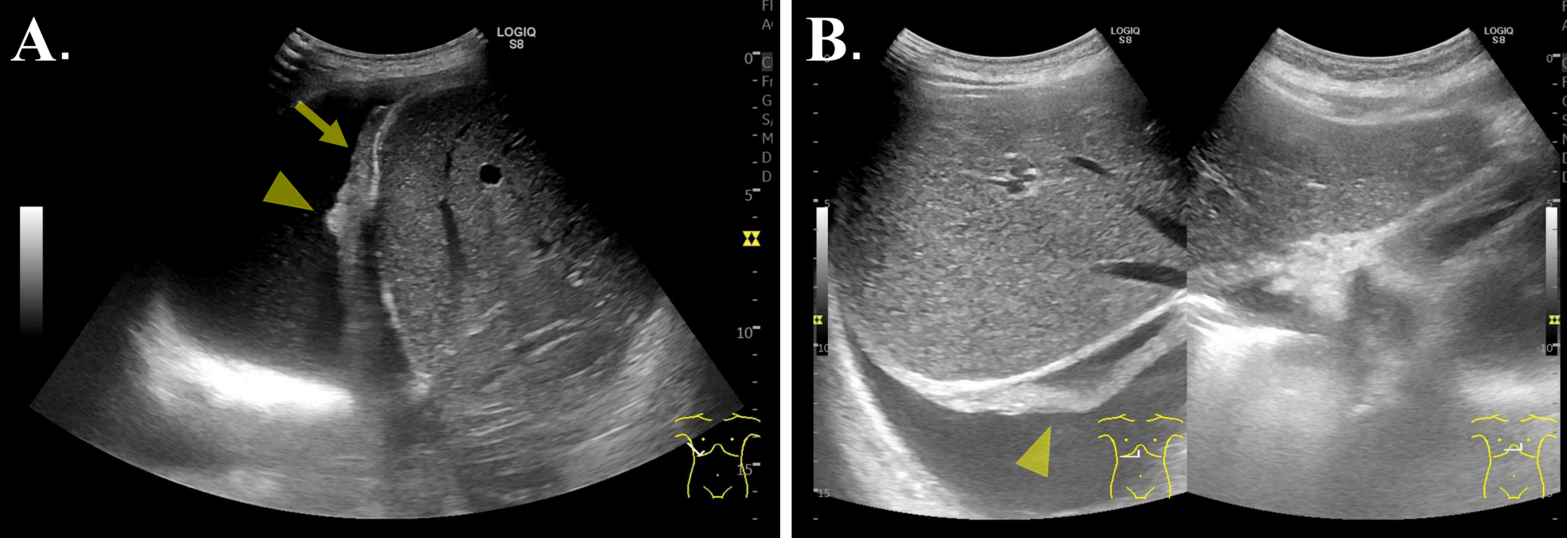
(A and B) Right pleural effusion accompanied by pleural thickening (arrow) and subpleural nodules (arrowhead) identified on lung ultrasound (with convex probe).

**Figure 2.**
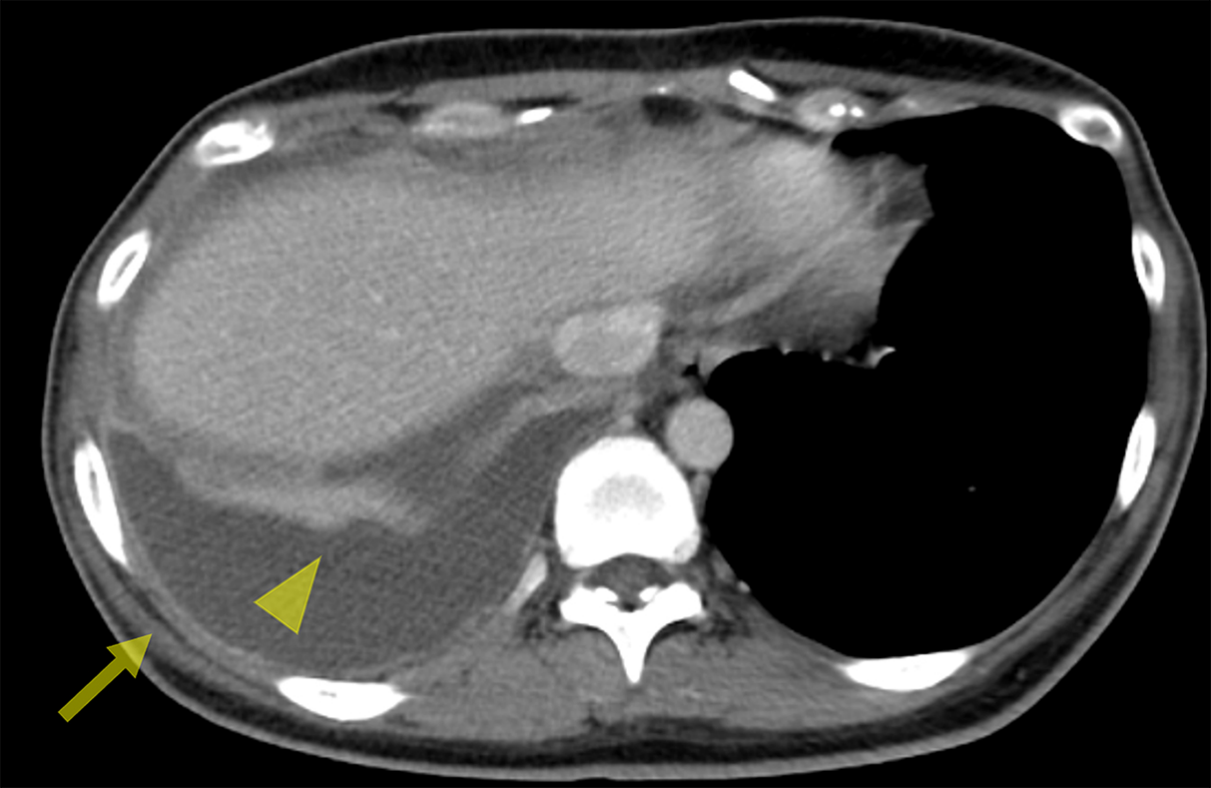
Right pleural effusion with enhanced pleural thickening (arrow) and subpleural nodules (arrowhead) in the right upper lobe on contrast-enhanced chest CT. CT: computed tomography.

Tuberculous pleurisy is not always easy to diagnose and may occur in the absence of pulmonary lesions. Inflammation of the peripheral diaphragmatic pleura, innervated by lower intercostal nerves, can cause referred pain in the lower chest or upper abdomen ^(1)^. While a definitive diagnosis of tuberculous pleurisy relies on pleural biopsy tissue samples, biopsy cultures, and pleural effusion cultures ^(2)^, lung ultrasound provides an accessible and noninvasive modality to support the diagnosis. Pleural thickness of approximately 3 mm is a frequently observed ultrasonographic finding in pleural tuberculosis ^(3)^. In adult tuberculous pleurisy, subpleural nodules are among the lung ultrasound findings with the highest sensitivities, ranging from 72.5% to 100% ^(4)^. The evaluation of the pleura should be incorporated into the workup of lateral abdominal pain, and lung ultrasound is a potentially valuable diagnostic tool.

## Article Information

### Author Contributions

According to the definition provided by the International Committee of Medical Journal Editors, the following individuals meet the criteria for authorship based on their substantial contributions to the intellectual content of the manuscript: Yuki Ohnishi: conceptualization, investigation, writing - original draft; Yasuhiro Suyama: investigation, writing - review and editing; Kenichi Nakamura: writing - review and editing. All authors have read and approved the final manuscript.

### Conflicts of Interest

None

### Ethical Approval and Consent to Participate

The authors obtained consent from the patients for the publication of this report, including images.

### Acknowledgment

The authors would like to express our gratitude to the support and feedback provided by Dr. Lance Bacon in the process of revising the manuscript.
